# Elongator subunit 3 positively regulates plant immunity through its histone acetyltransferase and radical *S*-adenosylmethionine domains

**DOI:** 10.1186/1471-2229-13-102

**Published:** 2013-07-16

**Authors:** Christopher T DeFraia, Yongsheng Wang, Jiqiang Yao, Zhonglin Mou

**Affiliations:** 1 Department of Microbiology and Cell Science, University of Florida, P.O. Box 110700, Gainesville, FL 32611, USA; 2 Interdisciplinary Center for Biotechnology Research, University of Florida, P.O. Box 103622, Gainesville, FL 32610, USA; 3 Current address: Department of Molecular Genetics, Ohio State University, Columbus, OH 43210, USA

**Keywords:** Arabidopsis, Elongator, Plant immunity, AtELP3, Transcription

## Abstract

**Background:**

Pathogen infection triggers a large-scale transcriptional reprogramming in plants, and the speed of this reprogramming affects the outcome of the infection. Our understanding of this process has significantly benefited from mutants that display either delayed or accelerated defense gene induction. In our previous work we demonstrated that the Arabidopsis Elongator complex subunit 2 (AtELP2) plays an important role in both basal immunity and effector-triggered immunity (ETI), and more recently showed that AtELP2 is involved in dynamic changes in histone acetylation and DNA methylation at several defense genes. However, the function of other Elongator subunits in plant immunity has not been characterized.

**Results:**

In the same genetic screen used to identify *Atelp2*, we found another Elongator mutant, *Atelp3-10*, which mimics *Atelp2* in that it exhibits a delay in defense gene induction following salicylic acid treatment or pathogen infection. Similarly to AtELP2, AtELP3 is required for basal immunity and ETI, but not for systemic acquired resistance (SAR). Furthermore, we demonstrate that both the histone acetyltransferase and radical *S*-adenosylmethionine domains of AtELP3 are essential for its function in plant immunity.

**Conclusion:**

Our results indicate that the entire Elongator complex is involved in basal immunity and ETI, but not in SAR, and support that Elongator may play a role in facilitating the transcriptional induction of defense genes through alterations to their chromatin.

## Background

Plants possess an innate immune system that protects them from microbial pathogens, but lack the adaptive immunity of mammals [1,2]. Each plant cell is capable of sensing non-self entities and mounting immune responses, demonstrating remarkable functional plasticity. Recognition of pathogen-associated molecular patterns (PAMPs) results in PAMP-triggered immunity (PTI) that prevents pathogen colonization. In turn, pathogens have evolved effectors to dampen PAMP-triggered signals and thereby attenuate PTI. The plants can only activate a weak response known as basal immunity. Some host plants have evolved resistance (R) proteins to detect the presence of pathogen effectors, inducing effector-triggered immunity (ETI) [3]. Activation of PTI or ETI leads to the generation of a blend of signal molecules, which move to distal tissues for the establishment of systemic acquired resistance (SAR). SAR is a long-lasting immunity against a broad spectrum of pathogens [4].

Salicylic acid (SA) is a key signal molecule for plant immunity against biotrophic and hemibiotrophic pathogens. It is not only required for the activation of SAR [5,6], but also plays an important role in plant basal immunity and ETI [7]–[11]. In plants, SA can be made through two metabolic pathways, the phenylalanine ammonia lyase (PAL)-mediated phenylalanine pathway and the isochorismate synthase (ICS)-mediated isochorismate pathway. Although knockout of *PAL* genes significantly reduces SA production [12], the isochorismate pathway is thought to be more important during plant defense [13]. Increasing cellular SA levels induces profound transcriptional changes that are largely governed by the transcription coactivator NPR1 (*n*onexpressor of *p*athogenesis-*r*elated (*PR*) genes). Similarly to SA, NPR1 is not only required for SAR activation, but also plays a significant role in plant basal immunity and ETI [7,14]–[17]. Interestingly, NPR1 is also a feedback inhibitor of SA biosynthesis. After pathogen infection, *npr1* plants accumulate significantly higher levels of SA [13,17]. Hyperaccumulation of SA causes chlorosis in juvenile leaves and inflorescences of *npr1* plants [18]. When grown on media containing high concentrations of SA, *npr1* seedlings fail to develop beyond the cotyledon stage, while wild type displays tolerance to SA cytotoxicity [19,20].

In eukaryotic cells, RNA Polymerase II (RNAPII) catalyzes the transcription of protein-encoding genes. The Elongator complex was first identified as an interactor of hyperphosphorylated RNAPII in yeast [21,22], and subsequently purified from mammalian and plant cells [23,24]. Elongator consists of six subunits (ELP1-6) with ELP1-3 forming the core subcomplex and ELP4-6 the accessory subcomplex [25,26]. Among the six subunits, ELP3 is the catalytic subunit, harboring a C-terminal histone acetyltranferase (HAT) domain and an N-terminal cysteine-rich motif that resembles an iron-sulfur (Fe-S) radical *S*-adenosylmethionine (SAM) domain [27,28]. ELP3 alone has intrinsic HAT activity and is capable of acetylating all four histones, whereas the six-subunit holo-Elongator predominantly acetylates lysine-14 of histone H3 and lysine-8 of histone H4 [22,27]. Consistently, the levels of acetylated histones H3 and H4 are reduced in yeast, mammalian, and plant *elp* mutants [24,27,29]. The radical SAM domain of yeast ELP3 is a structural motif required for the integrity of the complex [30], whereas the archaea *Methanocaldococcus jannaschii* ELP3 binds and cleaves SAM, a co-substrate involved in methyl group transfers, suggesting that *M*. *jannaschii* ELP3 may have another catalytic function other than HAT activity [31]. Indeed, a recent study indicated that the radical SAM domain of mouse ELP3, but not the HAT domain, is required for zygotic paternal genome demethylation [32].

Elongator is involved in diverse cellular processes including histone modification, tRNA modification, exocytosis, α-tubulin acetylation, and zygotic paternal genome demethylation [27,32,33]. Mutations in yeast Elongator subunits lead to resistance to the zymocin γ-toxin subunit, sensitivity to salt, caffeine and temperature [21,34,35]. Elongator deficiency in humans causes familial dysautonomia, an autosomal recessive disease, characterized by abnormally low numbers of neurons in the autonomic and sensory nervous systems [36,37]. In addition, Elongator has been shown to regulate tumorigenicity and migration of melanoma cells [38]. In plants, mutations of Elongator subunits result in pleiotropic effects including hypersensitivity to abscisic acid, resistance to oxidative stress, severely aberrant auxin phenotypes, disease susceptibility, and altered cell cycle progression [24,39]–[43].

In order to identify new components in SA signaling, we performed a genetic screen for suppressors of the *npr1* mutation based on restoration of SA tolerance on half-strength MS medium supplemented with 0.5 mM SA. A total of 20 *gns* (*g*reen n*pr1* seedling on *S*A medium) mutants showing restored SA tolerance have been isolated. We have previously described the *gns1* mutant, which harbors a mutation in AtELP2 [42]. Here we report the isolation and characterization of the *gns2* mutant, in which a frameshift mutation was identified in the Arabidopsis Elongator complex subunit 3 (AtELP3). Our results indicate that, like AtELP2, AtELP3 is required for plant basal immunity and ETI but not for SAR, and demonstrate that the HAT and radical SAM domains of AtELP3 are essential for its function in plant immunity.

## Results

### The *gns2* mutation suppresses hyperaccumulation of SA in *npr1*

**Figure 1:**
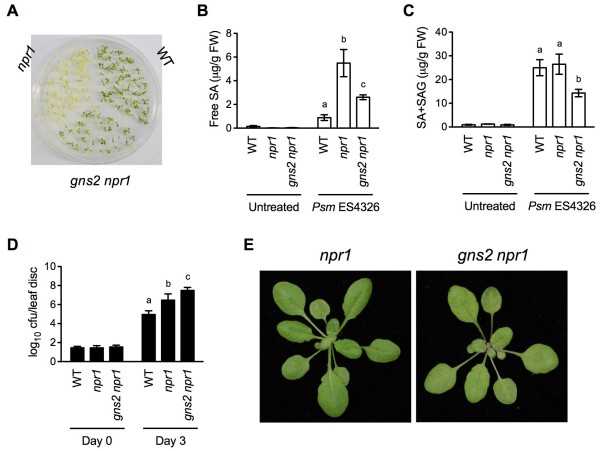
**Characterization of the *gns2 *Mutant. (A)** Seeds of wild type (WT), *npr1*, and *gns2 npr1* were placed on half-strength MS agar medium containing 0.26 mM SA. After three days of stratification, the plate was transferred to a growth chamber and photographed ten days later. **(B)** Four-week-old soil-grown WT, *npr1*, and *gns2 npr1* plants were inoculated with the virulent bacterial pathogen *Psm* ES4326 (OD_600_ = 0.001). Twenty-four hours later, the inoculated leaves were collected for SA measurement. Data represent the mean of three independent samples with standard deviation (SD). Different letters above the bars indicate significant differences (p < 0.05, Student’s *t*-test). **(C)** Total SA levels in *Psm* ES4326-infected WT, *npr1*, and *gns2 npr1* plants. The experiment was carried out as in (B). SAG, 2-*O*-β-D-glucosylsalicylic acid. **(D)** Leaves of four-week-old soil-grown WT, *npr1*, and *gns2 npr1* plants were inoculated with *Psm* ES4326 (OD_600_ = 0.0001). The *in planta* bacterial titers were determined immediately and three days postinoculation. Cfu, colony-forming units. Data represent the mean of eight independent samples with SD. Different letters above the bars indicate significant differences (p < 0.05, Student’s *t*-test). **(E)** Morphology of *npr1* and *gns2 npr1* plants. The plants were grown on soil for four weeks before the photos were taken. The *gns2 npr1* plant is a lighter shade of green than the *npr1* plant.

Similarly to the previously characterized *gns1 npr1*[42], the *gns2 npr1* mutant not only exhibited partially restored SA tolerance (Figure 1A), but also accumulated significantly less SA than *npr1* after infection by the virulent bacterial pathogen *Pseudomonas syringae* pv. *maculicola* (*Psm*) ES4326 (Figure 1B and C), suggesting that *gns2* suppresses SA hyperaccumulation in *npr1*. To test whether *gns2* affects pathogen susceptibility, the growth of *Psm* ES4326 was determined in *gns2 npr1* plants. As shown in Figure 1D, while *Psm* ES4326 grew ~32-fold more in *npr1* than in the wild type, its growth was further increased by ~10-fold in *gns2 npr1* plants, indicating that the *gns2* mutation compromises NPR1-independent disease resistance.

The leaves of *gns2 npr1* plants were a lighter shade of green than *npr1* (Figure 1E). The *gns2 npr1* mutant was backcrossed to *npr1* three times before further characterization. The F_1_ progeny were not SA tolerant and displayed *npr1* morphology. Of 72 F_2_ plants, 22 were *gns2*-like. The 1:3 *gns2*-like to *npr1*-like ratio (χ^2^ = 1.1852; P > 0.1), together with the F_1_ phenotypes fit the expectation that *gns2* is a single, recessive mutation. To determine the co-segregation of *gns2* morphology and SA tolerance, progeny from F_2_ plants with *npr1* or *gns2* morphology were examined. Progeny from *gns2*-like parents were nearly all SA tolerant, while none or only a fraction of the progeny from *npr1*-like plants were SA tolerant, suggesting that the morphology and SA tolerance of *gns2* co-segregate and are caused by the same mutation or two closely linked mutations.

### The *GNS2* locus encodes the Elongator subunit 3

**Figure 2:**
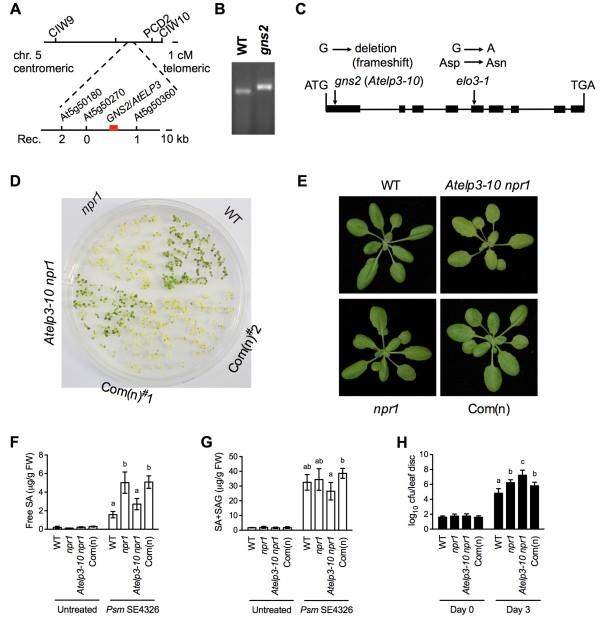
**Map-Based Cloning of *gns2. *(A)** Schematic representation of the mapping strategy for identifying the *gns2* mutation. New molecular markers used in this study are presented in Additional file 1: Table S1. **(B)** A dCAPS marker distinguishing *gns2* and WT. A deletion of a guanine (39 bp from ATG) was detected in the first exon of *AtELP3*, which allowed the development of a dCAPS marker to genotypically distinguish the mutant from the wild type (Additional file 1: Table S1). **(C)** Structure of the *GNS2*/*ELO3*/*AtELP3* gene and the positions of the *gns2* and *elo3-1* mutations [39]. Boxes denote the translated regions and lines between boxes denote introns. **(D)** Ten-day-old seedlings of WT, *npr1*, *Atelp3-10 npr1*, and two complementation lines Com(n)^#^1 and Com(n)^#^2 grown on half-strength MS agar medium containing 0.26 mM SA. Com(n), *35S::AtELP3 Atelp3-10 npr1* transgenic plants. **(E)** Four-week-old soil-grown WT, *npr1*, *Atelp3-10 npr1*, and Com(n) plants. (**F**) Free SA levels in *Psm* ES4326-infected WT, *npr1*, *Atelp3-10 npr1*, and Com(n) plants. Plants were inoculated with *Psm* ES4326 (OD_600_ = 0.001). Twenty-four hours later, the inoculated leaves were collected for SA measurement. Data represent the mean of four independent samples with SD. Different letters above the bars indicate significant differences (p < 0.05, Student’s *t*-test). **(G)** Total SA levels in *Psm* ES4326-infected plants. The experiment was carried out as in **(F)**. **(H)** Growth of *Psm* ES4326 in WT, *npr1*, *Atelp3-10 npr1*, and Com(n) plants. Cfu, colony-forming units. Leaves of four-week-old plants were inoculated with *Psm* ES4326 (OD_600_ = 0.0001). The *in planta* bacterial titers were determined immediately and three days postinoculation. Cfu, colony-forming units. Data represent the mean of eight independent samples with SD. Different letters above the bars indicate significant differences (p < 0.05, Student’s *t*-test).

To map the *GNS2* locus, homozygous *gns2 npr1*, which is in Columbia (Col-0) background, was crossed with the polymorphic ecotype Landsberg *erecta* (L*er*). Linkage analysis of 100 F_2_ plants with *gns2* morphology placed the *GNS2* locus in the middle of the lower arm of chromosome 5, between the molecular markers CIW9 and CIW10 (Figure 2A). Recombination analysis of 1352 F_2_ light green plants located the *GNS2* locus in between markers at the genetic loci At5g50180 and At5g50360 (Figure 2A). One gene within this interval was *AtELP3*/*ELONGATA3*(*ELO3*) (At5g50320) [39], which encodes the third subunit of the Elongator complex. Since *gns2* resembled the previously characterized *gns1*/*Atelp2-5* mutants in SA responses [42], *GNS2* might be *AtELP3*. The *AtELP3* coding region was therefore amplified from *gns2 npr1* and sequenced. A deletion of a guanine (39 bp from ATG) was detected in the first exon of *AtELP3*, which allowed the development of a dCAPS marker to genotypically distinguish the mutant from the wild type (Figure 2C and B and Additional file 1: Table S1). This mutation caused a frameshift, likely resulting in a truncated and non-functional protein.

To confirm that *GNS2* is *AtELP3*, the *AtELP3* cDNA under the control of the constitutive 35S promoter of cauliflower mosaic virus was introduced into *gns2 npr1*. The *35S::AtELP3* transgene complemented all the phenotypes caused by the *gns2* mutation in *gns2 npr1*, including partially restored SA tolerance, light green coloration, reduced SA accumulation after *Psm* ES4326 infection, and enhanced susceptibility to *Psm* ES4326 (Figure 2D to H). These results indicate that the mutation in *AtELP3* is responsible for the morphological and defense phenotypes of the *gns2 npr1* plants. The *gns2* mutant was therefore renamed *Atelp3-10*.

### AtELP3 positively contributes to salicylic acid responsiveness

**Figure 3:**
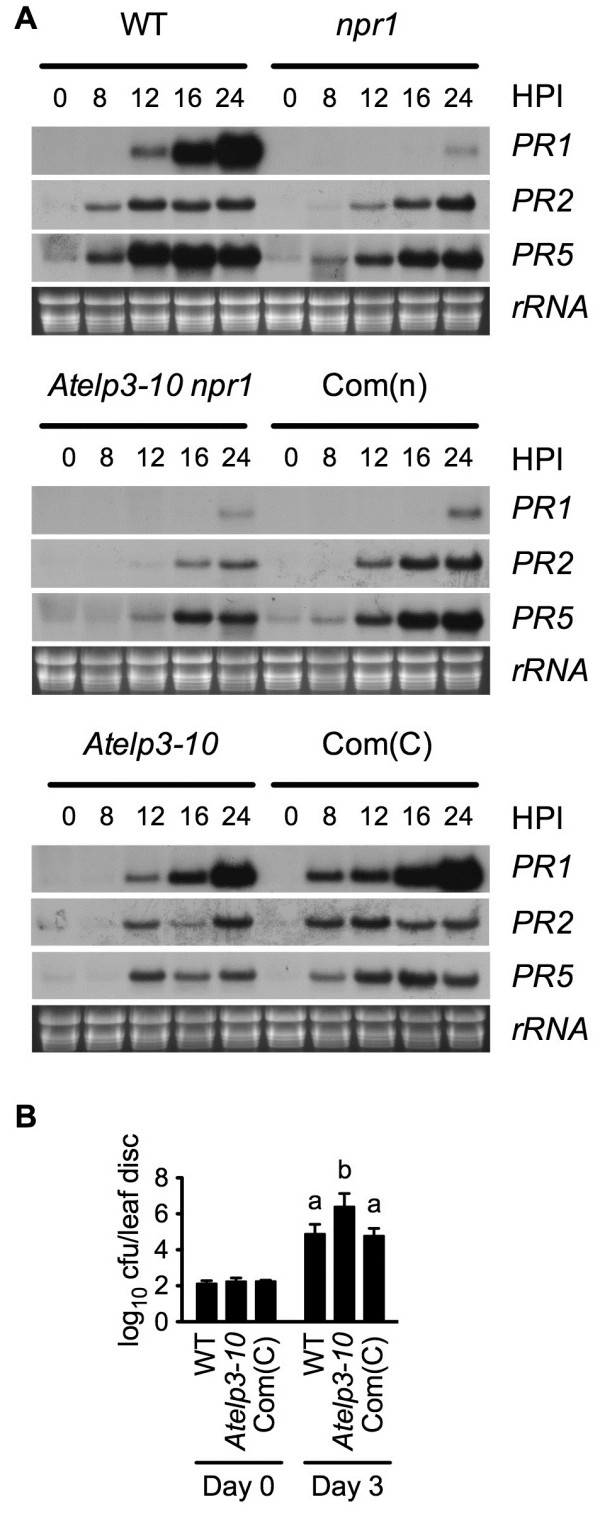
**Pathogen-Induced *PR *Gene Expression in *Atelp3-10 npr1 *and *Atelp3-10 *Plants. (A)** Expression of *PR* genes in *Psm* ES4326-infected WT, *npr1*, *Atelp3-10 npr1*, Com(n), *Atelp3-10*, and Com(C) plants. Com(n), *35S::AtELP3 Atelp3-10 npr1* transgenic plants; Com(C), *35S::AtELP3 Atelp3-10* transgenic plants. HPI, hours postinoculation. Plants were inoculated with *Psm* ES4326 (OD_600_ = 0.001). Leaf tissues were collected at the indicated time points after the inoculation. Total RNA was extracted and subjected to RNA gel blot analysis. The RNA samples were run on the same agarose gel and transferred onto the same blotting membrane. The rRNA bands in the ethidium bromide-stained gel were photographed as a loading control prior to blotting. **(B)** Growth of *Psm* ES4326 in WT, *Atelp3-10*, and Com(C) plants. Cfu, colony-forming units. Leaves of four-week-old plants were inoculated with *Psm* ES4326 (OD_600_ = 0.0001). The *in planta* bacterial titers were determined immediately and three days postinoculation. Data represent the mean of eight independent samples with SD. Different letters above the bars indicate significant differences (p < 0.05, Student’s *t*-test).

Compared with *npr1*, *Atelp3-10 npr1* exhibited delayed and reduced expression of *PR2* and *PR5* during *Psm* ES4326 infection (Figure 3A). To test the function of AtELP3 in the presence of NPR1, *Atelp3-10 npr1* was crossed with wild type, and *Atelp3-10* single mutants were identified in the segregating F_2_ population based on their morphology (same as the *Atelp3-10 npr1* double mutant) and confirmed by the *Atelp3-10* and *npr1* dCAPS markers (Additional file 1: Table S1). The *Atelp3-10* single mutant displayed decreased and/or delayed *PR* gene expression and enhanced susceptibility to *Psm* ES4326, and these phenotypes were all completely complemented by a *35S::AtELP3* transgene (Figure 3A and B), confirming that *Atelp3-10* is a loss-of-function mutation.

**Figure 4:**
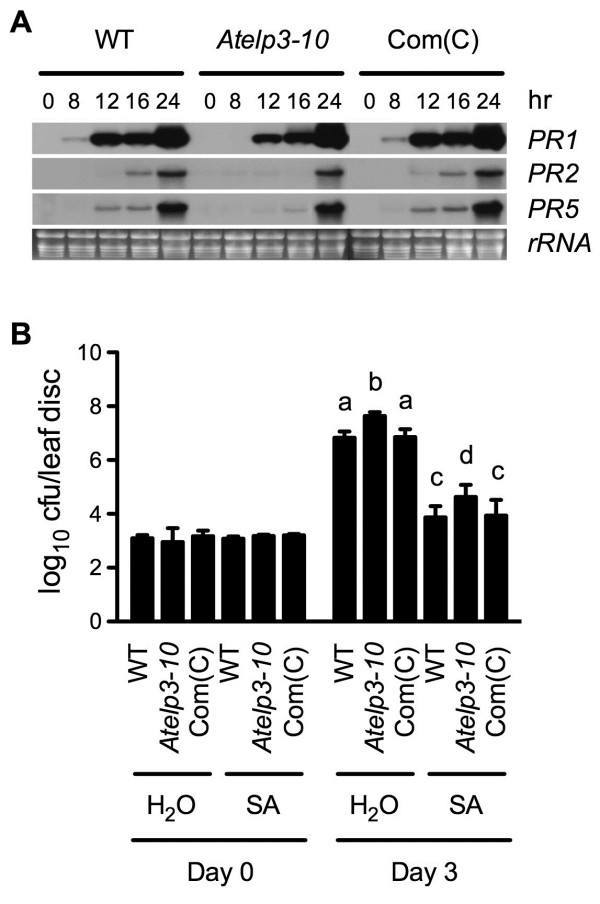
**SA-Induced *PR *Gene Expression and Resistance in *Atelp3-10. *(A)** Expression of *PR* genes in SA-treated WT, *Atelp3-10*, and Com(C) plants. Com(C), *35S::AtELP3 Atelp3-10* transgenic plants. Plants were treated with soil drenches of 1 mM SA water solution or water. Leaf tissue was collected at the indicated time points after the treatment. Total RNA was extracted and subjected to RNA gel blot analysis. The rRNA bands in the ethidium bromide-stained gel were photographed as a loading control prior to blotting. **(B)** Growth of *Psm* ES4326 in SA-treated WT, *Atelp3-10*, and Com(C) plants. Plants were treated with soil drenches of 1 mM SA solution or water. Twenty-four hours later, the plants were inoculated with *Psm* ES4326 (OD_600_ = 0.001). The *in planta* bacterial titers were determined immediately and three days postinoculation. Cfu, colony-forming units. Data represent the mean of eight independent samples with SD. Different letters above the bars indicate significant differences (p < 0.05, Student’s *t*-test).

When treated with SA, *Atelp3-10* exhibited delayed *PR* gene expression (Figure 4A), supporting the conclusion that Elongator plays a role downstream of SA in plant immune responses [42]. On the other hand, SA treatment substantially protected both *Atelp3-10* and wild type against *Psm* ES4326 (Figure 4B). We further analyzed the interaction between SA treatment and genotype with a linear mixed-effects model [44], and found that the *Atelp3-10* mutation did not significantly affect SA-induced resistance against *Psm* ES4326 (p = 0.8285).

### AtELP3 positively regulates effector-triggered immunity

**Figure 5:**
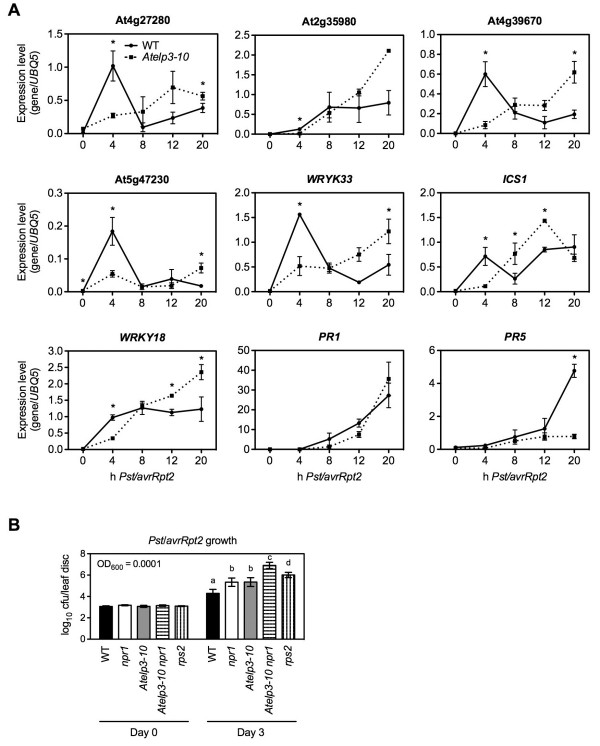
**Characterization of ETI in *Atelp3-10 *Plants. (A)** Expression of nine ETI-inducible genes in *Pst* DC3000/*avrRpt2*-infected WT and *Atelp3-10* plants. Plants were inoculated with *Pst* DC3000/*avrRpt2* (OD_600_ = 0.001). Leaf tissues were collected at the indicated time points. Total RNA was extracted and analyzed for the expression of indicated genes using real-time qPCR. The y-axes indicate relative expression levels monitored by qPCR. Expression was normalized against constitutively expressed *UBQ5*. The x-axes indicate hours after *Pst* DC3000/*avrRpt2* infection. Data represent the mean of three independent samples with SD. An asterisk (*) indicates that the expression level of the gene in the WT was significantly different from that in *Atelp3-10* (p < 0.05, Student’s *t*-test). **(B)** Growth of *Pst* DC3000/*avrRpt2* in WT, *npr1*, *Atelp3-10*, *Atelp3-10 npr1*, and *rps2* plants. Plants were inoculated with *Pst* DC3000/*avrRpt2* (OD_600_ = 0.0001). The *in planta* bacterial titers were determined immediately and three days postinoculation. Cfu, colony-forming units. Data represent the mean of eight independent samples with SD. Different letters above the bars indicate significant differences (p < 0.05, Student’s *t*-test).

To test whether AtELP3, like AtELP2, plays a role in ETI, we examined the expression profiles of nine defense genes that are rapidly induced during ETI [8,45]. As shown in Figure 5A, after infection with the ETI-inducing bacterial pathogen *P. s.* pv. *tomato* (*Pst*) DC3000/*avrRpt2*, induction of all nine genes was delayed in *Atelp3-10* compared with that in the wild type, though expression of some genes eventually reached wild-type levels. Furthermore, *Pst* DC3000/*avrRpt2* growth was ~10-fold more in *Atelp3-10* than in the wild type but lower than in the fully susceptible *rps2* mutant (Figure 5B) [46]. In the double mutant *Atelp3-10 npr1*, however, *Pst* DC3000/*avrRpt2* growth was ~35-fold higher than in either *Atelp3-10* or *npr1*, and about sevenfold higher than in *rps2* (Figure 5B). These results are consistent with the conclusion that both Elongator and NPR1 positively regulate ETI [42]. Using a linear mixed-effects model to statistically analyze the data in Figure 5B, we found that the *Atelp3-10* and *npr1* mutations did not have statistically significant genetic interaction in resistance against *Pst* DC3000/*avrRpt2* (p = 0.064); therefore, AtELP3 and NPR1 appear to function largely independently of each other in ETI-mediated resistance to *Pst* DC3000/*avrRpt2*. However, since the p value is very close to the significance threshold (0.05), we cannot rule out the possibility of genetic interaction between AtELP3 and NPR1. Indeed, our recent work indicated that AtELP2 regulates both NPR1-dependent and -independent defense gene induction [47].

### AtELP3 is not required for biological induction of systemic acquired resistance

**Figure 6:**
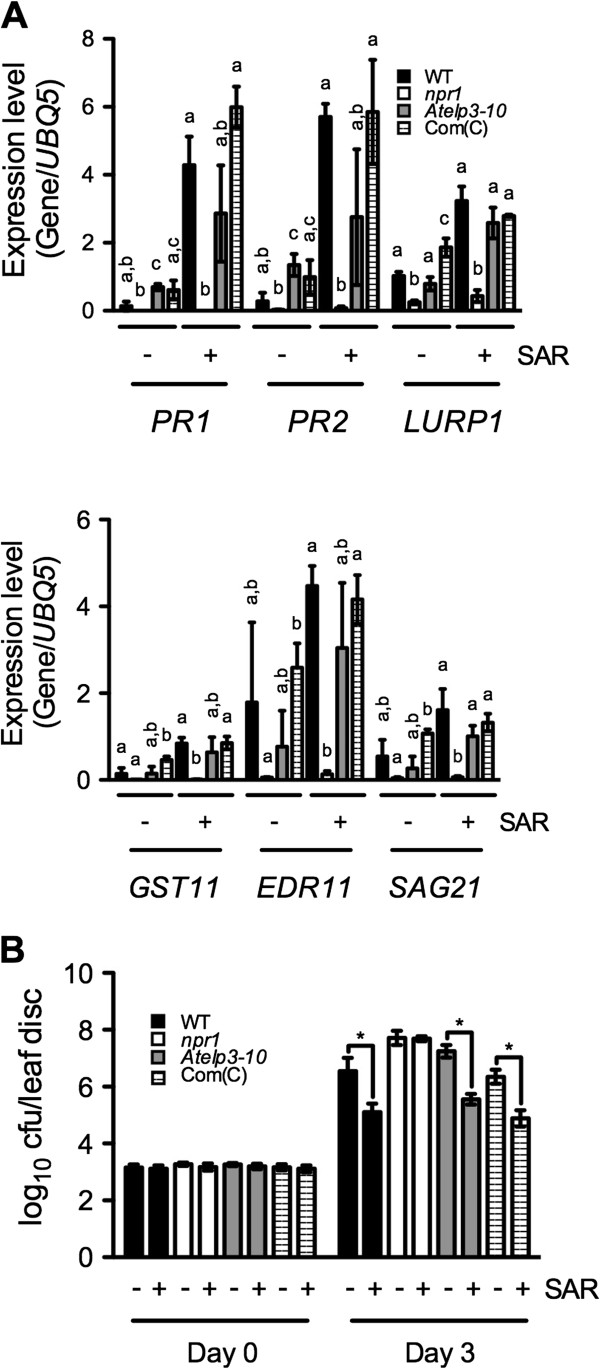
**SAR Induction in *Atelp3-10 *Plants. (A)** Expression of six SAR-associated genes in systemic leaves of WT, *npr1*, *Atelp3-10*, and Com(C) plants. Com(C), *35S::AtELP3 Atelp3-10* transgenic plants. Three lower leaves on each plant were inoculated with *Psm* ES4326 (OD_600_ = 0.002) (+SAR) or mock-treated with 10 mM MgCl_2_ (−SAR). Two days later, total RNA was extracted from the upper uninfected/untreated leaves and analyzed for the expression of indicated genes by real-time qPCR. Expression was normalized against constitutively expressed *UBQ5*. Data represent the mean of three independent samples with SD. Different letters above the bars indicate significant differences (p < 0.05, Student’s *t*-test). Note that the comparison was made among WT, *npr1*, *Atelp3-10*, and Com(C) for each gene/treatment. **(B)** SAR induction in WT, *npr1*, *Atelp3-10*, and Com(C) plants. Three lower leaves on each plant were inoculated with *Psm* ES4326 (OD_600_ = 0.002) (+SAR) or mock-treated with 10 mM MgCl_2_ (−SAR). Two days later, two upper uninfected/untreated leaves were challenge-inoculated with *Psm* ES4326 (OD_600_ = 0.001). The *in planta* bacterial titers were determined immediately and three days after challenge inoculation. Cfu, colony-forming units. Data represent the mean of ten independent samples with SD. *Psm* ES4326 grew significantly less in the SAR-induced WT, *Atelp3-10*, and Com(C) plants than in the mock-treated plants (*All p < 0.05, Student’s *t*-test).

We next analyzed the induction of SAR in *Atelp3-10* plants. Three lower leaves were injected with 10 mM MgCl_2_ (mock treatment) or *Psm* ES4326 (SAR treatment). After two days, SAR treatment-induced gene expression in the upper, untreated leaves was examined. Six genes that are induced during SAR [48] were strongly NPR1-dependent, but only required AtELP3 for their full expression (Figure 6A). Expression levels of these genes were significantly higher in *Atelp3-10* plants than in *npr1* plants (Figure 6A). When the upper, untreated systemic leaves were challenge-inoculated with *Psm* ES4326, *Atelp3-10* exhibited similar levels of resistance as the wild type (Figure 6B). Using a linear mixed-effects model to statistically analyze the data in Figure 6B, we found that the *Atelp3-10* mutation did not have statistically significant effect on SAR treatment-induced resistance to *Psm* ES4326 (p = 0.279). Therefore, SAR induction restores pathogen resistance to *Atelp3-10* plants, which supports the conclusion that Elongator is not required for SAR activation [42].

### Both HAT and radical SAM domains of AtELP3 are required for its function in plant immunity

**Figure 7:**
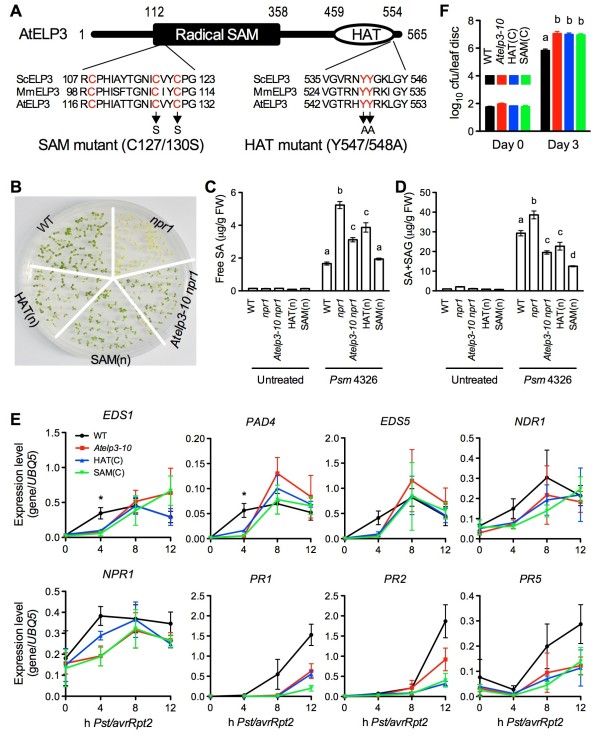
**Characterization of the HAT and Radical SAM Domain Mutants of AtELP3. (A)** Schematic representation of the HAT and radical SAM domain mutations. The radical SAM and HAT domains of AtELP3 are aligned with those of the yeast (ScELP3) and mouse ELP3 (MmELP3). Only sequences that are part of the alignment are shown. The conserved amino acid residues are labeled in red. Arrows indicate the mutations created in the SAM and HAT mutants. **(B)** Ten-day-old seedlings of WT, *npr1*, *Atelp3-10 npr1*, HAT(n), and SAM(n) grown on half-strength MS agar medium containing 0.26 mM SA. HAT(n) and SAM(n), transgenic lines in the *Atelp3-10 npr1* genetic background expressing the HAT and the SAM mutant, respectively. **(C)** Free SA levels in *Psm* ES4326-infected WT, *npr1*, *Atelp3-10 npr1*, HAT(n), and SAM(n) plants. Plants were inoculated with *Psm* ES4326 (OD_600_ = 0.001). Twenty-four hours later, the inoculated leaves were collected for SA measurement. Data represent the mean of three independent samples with SD. Different letters above the bars indicate significant differences (p < 0.05, Student’s *t*-test). **(D)** Total SA levels in *Psm* ES4326-infected WT, *npr1*, *Atelp3-10 npr1*, HAT(n), and SAM(n) plants. Experiment was performed as in (C). **(E)** Expression of eight defense genes in *Pst* DC3000/*avrRpt2*-infected WT, *Atelp3-10*, HAT(C), and SAM(C) plants. HAT(C) and SAM(C), transgenic lines in the *Atelp3-10* genetic background expressing the HAT and the SAM mutant, respectively. Data represent the mean of three independent samples with SD. An asterisk (*) indicates that the expression level of the gene in the WT was significantly higher than that in *Atelp3-10*, HAT(C), and SAM(C) (p < 0.05, Student’s *t*-test). **(F)** Growth of *Psm* ES4326 in WT, *Atelp3-10*, HAT(C), and SAM(C) plants. Cfu, colony-forming units. Data represent the mean of eight independent samples with standard deviation. Different letters above the bars indicate significant differences (p < 0.05, Student’s *t*-test).

ELP3 is the catalytic subunit of the Elongator complex, containing a HAT domain and a radical SAM domain. Both domains are required for Elongator’s function in yeast, whereas only the radical SAM domain is essential for mouse paternal genome demethylation in zygotes [32]. To test whether the HAT and the radical SAM domain of AtELP3 are required for Elongator’s function in plant immunity, we introduced point mutations into the *AtELP3* cDNA to generate a HAT domain mutant, in which two conserved tyrosine residues (Y547 and Y548) were changed to alanine, and a radical SAM domain mutant, in which two conserved cysteine residues (C127 and C130) were changed to serine (Figure 7A) [32]. Mutated *Atelp3* cDNAs were put under the control of the constitutive 35S promoter and transformed into *Atelp3-10* and *Atelp3-10 npr1* to test the functionality of the HAT and the SAM mutant. Multiple transgenic lines were generated and characterized for both constructs, and one representative transgenic line for each construct was shown in Figure 7. Neither the HAT nor the SAM mutant complemented any of the *Atelp3-10* phenotypes, including partially restored SA tolerance (Figure 7B), reduced SA accumulation after *Psm* ES4326 infection (Figure 7C and D), delayed/decreased defense gene expression (Figure 7E), and enhanced susceptibility to *Psm* ES4326 (Figure 7F), indicating that both the HAT and radical SAM domains of AtELP3 are essential for its function in plant immune responses.

## Discussion

The multiprotein complex Elongator functions in diverse biological processes in yeast, animals, and plants likely by accelerating the induction of genes required for development or adaptation to changing environmental conditions. In Arabidopsis, Elongator regulates transcriptional changes induced during pathogen infection [42,47]. These transcriptional changes are accompanied by changes in DNA methylation, a hallmark of transcriptional suppression [49], and histone acetylation, which is generally associated with transcriptional activation [47,50]. Since the Elongator complex subunit AtELP3 contains both HAT and radical SAM domains, it seems possible that the enzymatic activities of Elongator are directly involved in altering the chromatin status of defense genes to facilitate their induction. Fortuitously, we were able to isolate an *Atelp3* mutant in our genetic screen for suppressors of the *npr1* mutation based on restoration of SA tolerance (Figures 1 and 2) [42]. The *Atelp3-10* mutant displays similar deficiencies in basal immunity and ETI as *Atelp2* (Figures 1D, 2H, and 5B), and like *Atelp2* has normal SAR (Figure 6B), suggesting that the Elongator complex itself, but not AtELP2 individually, is the functional unit of action for its role in plant immunity. Indeed, T-DNA insertion mutants of other subunits of the Arabidopsis Elongator complex exhibit *Atelp2*- and *Atelp3*-like morphology [39,41]. The function of these subunits in plant immunity will be a subject of further inquiry.

Transgenic complementation of *Atelp3-10* with the *AtELP3* gene lacking conserved residues in the HAT domain did not restore wild-type levels of SA tolerance, SA accumulation, defense gene induction, and pathogen resistance (Figure 7). Although Elongator’s HAT activity has not been demonstrated in plants, it has been proven in yeast [27]. Consistent with this, the levels of acetylated histones are reduced in yeast, mammalian, and plant *elp* mutants [24,27,29]. Previously we found reduced histone acetylation levels at several defense genes in *Atelp2*, which was correlated with delayed and/or decreased induction of these genes [47]. Taken together, these results suggest that Elongator-dependent histone acetylation may maintain an inducible state at defense loci in Arabidopsis.

Conserved residues in the radical SAM domain proved equally important for AtELP3’s function in plant immunity (Figure 7). Although these data demonstrate the importance of the domain, since the enzymatic activity of this domain is currently unknown, the reason for this importance remains elusive. One possibility is that the radical SAM domain is important for the structural integrity of the protein complex, and in yeast, this has been shown to be the case [30]. Alternatively, ELP3 may bind and cleave SAM, as seen in the archaea *M. jannaschii*[31]. Indeed, a recent study indicated that the radical SAM domain of mouse ELP3, but not the HAT domain, is required for zygotic paternal genome demethylation [32]. Previously we found altered dynamic DNA methylation changes induced by pathogen infection at some defense loci in *Atelp2*, which was also correlated with delayed and/or decreased induction [47]. Therefore, Elongator may also modulate chromatin at defense loci in Arabidopsis through DNA methylation.

Another, non-mutually exclusive possibility is that the role of Elongator in plant immunity depends on its function in the modification of wobble uridine tRNA. In yeast and plants, Elongator is essential for these modifications [51,52]. Using overexpression of hypomodified uridine-containing tRNAs, all phenotypes of yeast *elp* mutants were shown to stem from reduced formation of fully modified tRNAs [53]. Most yeast mutations in the radical SAM domain result in complete loss of the modified nucleoside 5-methoxycarbonylmethyl-2-thiouridine (mcm^5^s^2^U), while mutations of the HAT domain result in the loss of the majority of mcm^5^s^2^U. Loss of mcm^5^s^2^U is closely correlated with the *elp* mutant phenotypes [53]. In yeast, mutations of conserved amino acids in the SAM domain do not abrogate HAT or RNA binding activity [30]; therefore, for all Elongator roles examined including plant immunity, with the exception of zygotic paternal genome demethylation [32], both the HAT and radical SAM domains of ELP3 are essential, but neither is sufficient. Since plant Elongator is also essential for wobble uridine tRNA modifications [52], it is possible that the immune phenotypes, as well as other *elp* phenotypes, including altered histone modification and DNA methylation profiles, could be indirect effects of tRNA modification defects in these mutants. Future investigations in plants should address this important question.

Accumulating evidence from diverse organisms is starting to paint a picture where the transcriptional role of Elongator is to facilitate the induction of genes in general. Indeed, previous work performed in our lab and elsewhere has shown that Elongator influences the inducibility of thousands of genes [35,47,54]. In this study we established that the HAT and radical SAM domains of the Arabidopsis Elongator subunit AtELP3 are essential for Elongator’s immune function. Elongator has also been implicated in diverse stress responses including oxidative and drought stress in Arabidopsis [41]. It would be interesting to determine the role of the HAT and radical SAM domains of AtELP3 in these processes. In this regard, the HAT and radical SAM domain mutants generated in this study provide a useful tool for dissecting the role of these domains throughout development and under diverse stress conditions.

## Conclusions

In this study, we isolated the *gns2* mutant and cloned the *GNS2* gene using a map-based cloning approach. We found that *GNS2* encodes the Elongator subunit AtELP3. Characterization of *gns2*/*Atelp3-10* indicates that AtELP3 is required for basal immunity and ETI, but not for SAR, suggesting that Elongator as a whole is likely involved in basal immunity and ETI, but not in SAR. Furthermore, we demonstrate that AtELP3’s function in plant immunity requires both the HAT and radical SAM domains, which is consistent with a role of Elongator in facilitating the transcriptional induction of defense genes through alterations to their chromatin.

## Methods

### Plant materials and pathogen infection

The wild type used was the *Arabidopsis thaliana* (L.) Heynh. Columbia (Col-0) ecotype, and the mutant alleles used were *npr1-3*[19] and *rps2-201C*[46]. Arabidopsis seeds were sown on autoclaved soil (Metro-Mix 200, Grace-Sierra, Malpitas, CA) and vernalized at 4°C for three days. Plants were germinated and grown at ~23°C under a 16-hr-light/8-hr-dark regime.

Inoculation of plants with *Psm* ES4326 and *Pst* DC3000/*avrRpt2* was performed by pressure-infiltration with a 1 mL needleless syringe as described previously [18]. After inoculation, eight infected leaves, one from each plant, were collected for each genotype, treatment or time point to determine *in planta* growth of the pathogen. For SAR induction, three lower leaves on each plant were inoculated with the virulent bacterial pathogen *Psm* ES4326 (OD_600_ = 0.002). Two days later, the upper uninfected systemic leaves were either collected for gene expression analysis or challenge-inoculated with *Psm* ES4326 (OD_600_ = 0.001) for resistance test. Ten leaves were collected three days after challenge inoculation to examine the pathogen growth.

### RNA analysis

RNA extraction and RNA gel blot analysis were carried out as described by Cao et al. [14] and Glazbrook et al. [55]. For reverse transcription (RT), total RNA was treated with DNase I (Gibco, BRL) at 37°C for 30 min. After inactivation of the DNase, RT was performed using SUPERSCRIPT First-Strand Synthesis System (Gibco, BRL) and 2 μg of the DNase-treated RNA in a 20 μL reaction. Aliquots of the resulting RT reaction product were used for real-time quantitative PCR (qPCR), which was performed using SYBR Green protocol (Applied Biosystems, Foster City, CA) with 1 μM primers and 0.2 μL aliquot of RT product in a total of 12.5 μL per reaction. Reactions were run and analyzed on a MX3000P real-time PCR machine (Stratagene, La Jolla, CA) according to the manufacturer’s instructions. The relative quantity of the tested gene is expressed in relation to ubiquitin (*UBQ5*) using the formula 2^(Ct{*UBQ5*}-Ct{*GENE*}), where 2 represents perfect PCR efficiency. We chose *UBQ5* as the reference gene for qPCR normalization, because it is one of the most stably expressed genes [56]. All primers used for qPCR in this study have been reported in DeFraia et al. [42].

### SA measurement

SA measurement was done with HPLC as described by Verberne et al. [57].

### Plasmid construction and plant transformation

A pair of primers *BamH I-AtELP3F1* (5′-CGGGATCCATGGCGACGGCGGTAGTGATG-3′) and *Sac I-AtELP2R* (5′-CCGAGCTCTCAAAGAAGATGCTTCACCATGTAAG-3’) was used to amplify the coding region of *AtELP3* from total cDNA generated by RT. The PCR products were digested with BamH I and Sac I and then ligated into the corresponding sites of the vector pBI1.4 T, resulting in the plamsid pBI1.4 T-35S::AtELP3. Site-directed mutagenesis of the conserved amino acid residues in the HAT and radical SM domains of AtELP3 was performed in the pBI1.4 T-35S::AtELP3 construct using a PCR-based Quick-Change site-directed mutagenesis kit (Stratagene, La Jolla, CA). The primers used for the site-directed mutagenesis of the HAT domain were *AtELP3-HATMuF* (5′-GTAGGAACCAGACATGCCGCCAGAAAGTTGGGTTATG-3′) and *AtELP3-HATMuR* (5′-CATAACCCAACTTTCTGGCGGCATGTCTGGTTCCTAC-3′), and for the radical SAM domain mutagenesis, the primers were *AtELP3-SAMMuF* (5′-CGACGGGGAATATATCCGTTTATTCTCCCGGTGGACCTGAC-3′) and *AtELP3-SAMMuR* (5′-GTCAGGTCCACCGGGAGAATAAACGGATATATTCCCCGTCG-3′). The presence of the expected mutations in the resulting construct was verified by DNA sequencing. The plasmids were introduced into the Agrobacterium strain GV3101(pMP90) by electroporation [58], and transformation was performed following the floral dip method [59].

### Statistical methods

Statistical analyses were performed with the data analysis tools (Student’s *t*-test: Two Samples Assuming Unequal Variances) in Microsoft Excel of Microsoft Office 2004 for Macintosh. Linear mixed-effects model analysis was performed with the software SAS 9.3. All experiments were repeated at least three times with similar results.

## Abbreviations

AtELP3: Arabidopsis Elongator complex subunit 3; ELO3: ELONGATA3; HAT: histone acetyltransferase; SAM: *S*-adenosylmethionine; SA: salicylic acid; PAMPs: pathogen-associated molecular patterns; PTI: PAMP-triggered immunity; ETI: effector-triggered immunity; npr1: *n*onexpressor of *p*athogenesis-*r*elated (*PR*) genes 1; gns: *g*reen n*pr1* seedling on *S*A medium; SAR: systemic acquired resistance; ICS: isochorismate synthase; PAL: phenylalanine ammonia lyase; RNAPII: RNA Polymerase II; Psm: *Pseudomonas syringae* pv.* maculicola.*

## Competing interests

The authors declare that they have no competing interests.

## Authors’ contributions

CTD and ZM initiated the research. CTD and YW carried out the molecular experiments. JY helped with data analysis. CTD and ZM wrote the manuscript. All authors critically read and approved the final version of the manuscript.

## Supplementary Material

Additional file 1: Table S1 New (d)CAPS markers used in this study. Click here for file
