# Supplementary material for: Elongator subunit 3 positively regulates plant immunity through its histone acetyltransferase and radical S-adenosylmethionine domains
Source: BMC Plant Biol. 2013 Jul 16;13:102. doi: 10.1186/1471-2229-13-102 (PMC3728140; doi:10.1186/1471-2229-13-102)
Supplement: Additional file 1: Table S1 — New (d)CAPS markers used in this study. [file 1471-2229-13-102-S1.doc]

**SUPPLEMENTAL TABLES**

**Supplemental Table 1.** New (d)CAPS markers used in this study

| Marker name | Forward primer (5’ to 3’) | Reverse primer (5’ to 3’) | Restriction enzyme | Col (bp) | L*er* (bp) |
| --- | --- | --- | --- | --- | --- |
| At5g47570 | CCACTCAACCATGCCAATGC | CGTCGGCAAACACATCGTCC | ApoI | 250 | 211, 39 |
| At5g50270 | CGGTTTGTTGACAGATCTTTG | CATATCCTGCATACAAGACAG | BstUI | 758 | 521, 237 |
| At5g50120 | TTACCAAAGATTTCACTGCTCC | CATCCTACTCATCATCCCATC | BbsI | 500 | 248, 252 |
| At5g50180 | GAACCATGTGATATGTTTCACC | GCAAGATTACTTCAAACCTTGC | BtsCI | 604 | 372, 232 |
| At5g50210 | TTGTTGGGTGGAGAGAGATC | CCAGCTTGATCTAGGATGGC | RsaI | 793 | 463, 330 |
| At5g50270 | CGGTTTGTTGACAGATCTTTG | CATATCCTGCATACAAGACAG | BstUI | 758 | 521, 237 |
| At5g50300 | TAGAAACAATGCATGCACAAGC | AGTGGTGTGTGAGTGTGTACCG | BsrI | 228 | 197, 31 |
| At5g50360 | ACCACCAGATCCGTTCTTCG | CACATCCAAAGGAGATTCGTG | EcoRV | 782 | 540, 242 |
| At5g50390 | CCACAAGAACACTTCCCTTC | GGCAGATATACCAAAAAGTGG | Tsp509I | 318 | 216, 102 |
| At5g50460 | CCATTATTGCTCGTTAGTTAC | TGGGATGCAATATTTTGGCC | BslI | 279 | 211, 68 |
| At5g50770 | CTAAGAGCCTCCATCATTGG | GCAAGTTCATGACAAAGGAC | DpnII | 586 | 483, 103 |
| At5g51130 | GCCTTACAGGAGGTTTCTAG | GGTCGTAAAACAATCGTTCG | ApoI | 477 | 288, 189 |
| *PDC2* | CAGTGGATCACTCCCAAGACGCCTC | GCACTCAACTTATATATATTTCAG | BamHI | 425 | 360, 65 |
| *Atelp3-10* (dCAPS) | CCTTTACCTGGCCGAGGTTG | CATTTTATGTCTTTTCTGTTGCCTC | MwoI | 192 | 225 |
